# An Aggressive Cerebral Cavernous Malformation Presenting With Hemorrhage: A Case Report

**DOI:** 10.7759/cureus.92776

**Published:** 2025-09-20

**Authors:** Yuki Hayashi, Shusaku Matsuo, Ryusei Seo, Takumi Kitamura, Takeshi Torigai

**Affiliations:** 1 Neurosurgery, Chutoen General Medical Center, Kakegawa, JPN

**Keywords:** aggressive, cerebral cavernous malformations, hemorrhage, increase in size, rebleeding, vascular malformation

## Abstract

Cerebral cavernous malformations (CCMs) are rare vascular malformations that are typically not visualized on cerebral angiography and are often discovered incidentally on brain MRI. However, it may also present symptomatically with seizures or intracerebral hemorrhage. We report the case of a 23-year-old woman in whom a hemorrhagic CCM located in the right temporal lobe was identified following a 10-day history of headache accompanied by nausea and vomiting. Initial management involved conservative observation. However, within approximately one month, the lesion demonstrated two episodes of asymptomatic rebleeding and an increase in size from 33.8 mm to 46.8 mm in maximum diameter, indicating highly aggressive behavior. Given the potential risk of neurological deterioration or even fatal outcomes from further hemorrhages, surgical resection was performed. The patient was discharged without any neurological deficits and is scheduled for follow-up with outpatient MRI surveillance.

## Introduction

CCMs are rare vascular anomalies that are typically not detectable by conventional angiography and are often identified incidentally on MRI [1]. Although many CCMs remain asymptomatic, they can sometimes cause intracerebral hemorrhage. Among hemorrhagic CCMs, a subset is prone to rebleeding, with an estimated annual risk of approximately 4.5% [2]. The median interval to rebleeding has been reported to range from eight to 10 months [3, 4]. However, cases involving multiple rebleeding events within a short period are exceedingly rare, and clinical management in such situations remains challenging. In this report, we present the case of a young patient with a CCM that exhibited a highly aggressive clinical course, characterized by at least two rebleeding episodes within approximately one month and rapid lesion enlargement. We also discuss considerations regarding appropriate clinical management in such atypical and rapidly progressive cases.

## Case presentation

**Figure 1:**
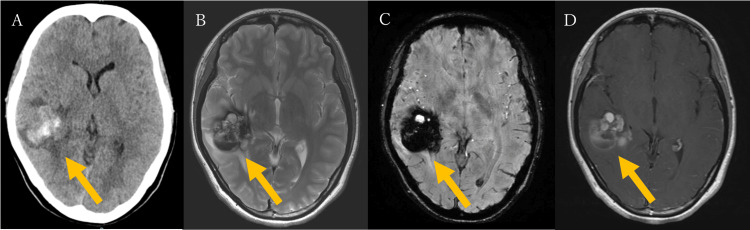
The patient's initial head CT and MRI findings (A) Non-contrast head CT reveals an intracerebral hemorrhage in the right temporal lobe with surrounding brain edema. (B) T2-weighted MRI shows a popcorn-like lesion with a surrounding hypointense rim. (C) Susceptibility-weighted imaging (SWI) demonstrates blooming artifacts around the lesion. (D) Gadolinium-enhanced MRI shows partial contrast enhancement of the lesion.

A 23-year-old woman presented to the internal medicine department with a 10-day history of headache, nausea, and vomiting. Neurological examination revealed no abnormalities; however, a non-contrast head CT scan performed at presentation revealed an intracerebral hemorrhage in the right temporal lobe, accompanied by surrounding brain edema (Figure 1A). The patient was promptly referred to neurosurgery.

**Figure 2:**
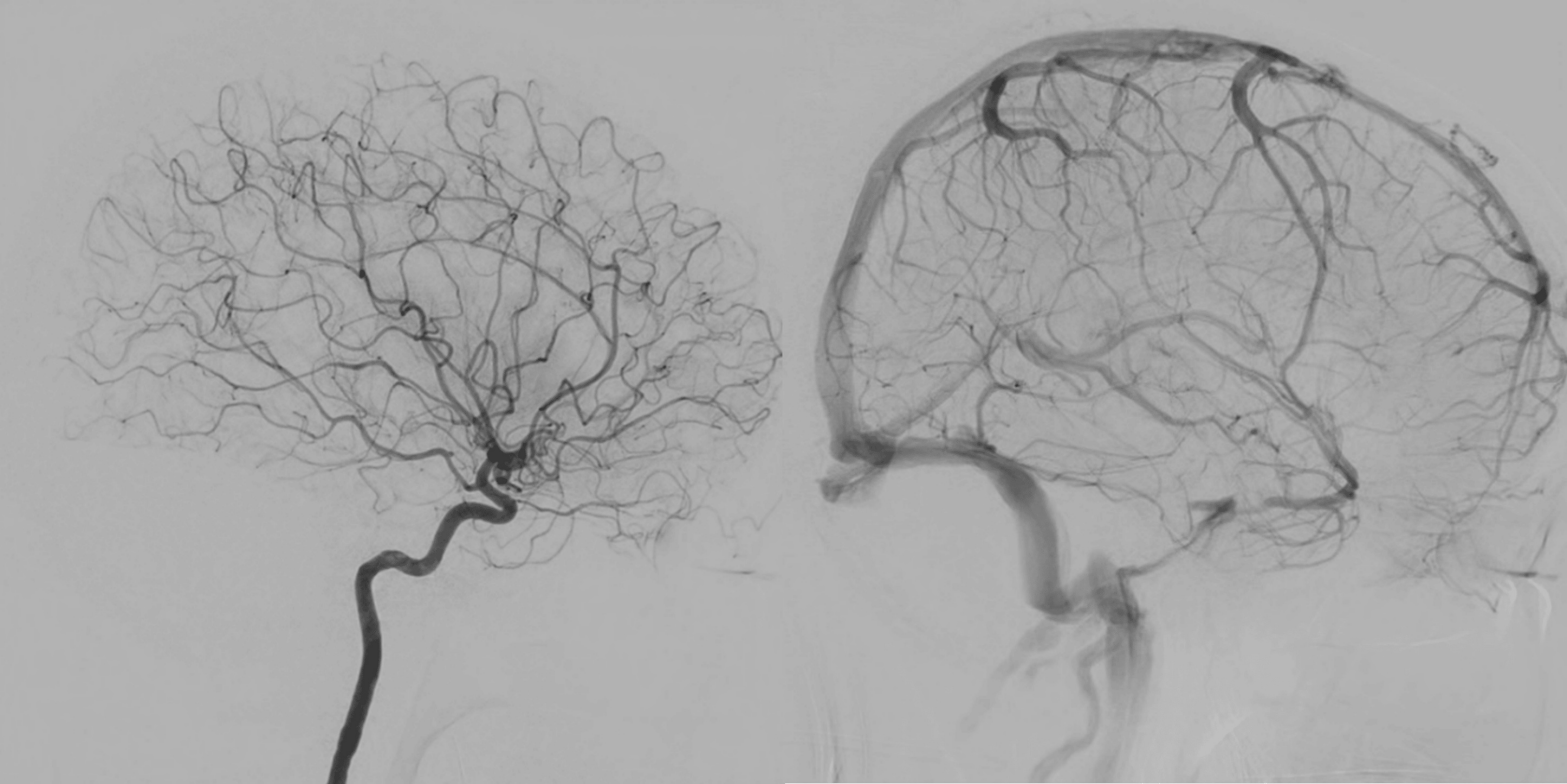
Digital subtraction angiography (DSA) of the right internal carotid artery DSA of the right internal carotid artery shows no abnormal contrast enhancement corresponding to the lesion. No evidence of arteriovenous shunting or developmental venous anomalies (DVAs) is observed.

The patient had no significant medical history apart from migraines and denied any recent head trauma. She was admitted for conservative management and further evaluation of the hemorrhage source. On hospital day 2, brain MRI revealed a well-circumscribed, round, popcorn-like lesion of variable signal intensities in the right temporal lobe on T2-weighted imaging (Figure 1B). Susceptibility-weighted imaging (SWI) demonstrated blooming artifacts consistent with hemorrhage (Figure 1C). The maximum diameter of the lesion was 33.8 mm. A gadolinium-enhanced MRI showed partial enhancement, but no developmental venous anomalies (DVAs) were identified (Figure 1D). Digital subtraction angiography (DSA) performed on hospital day 9 similarly showed no evidence of DVAs or shunting disease (Figure 2).

**Figure 3:**
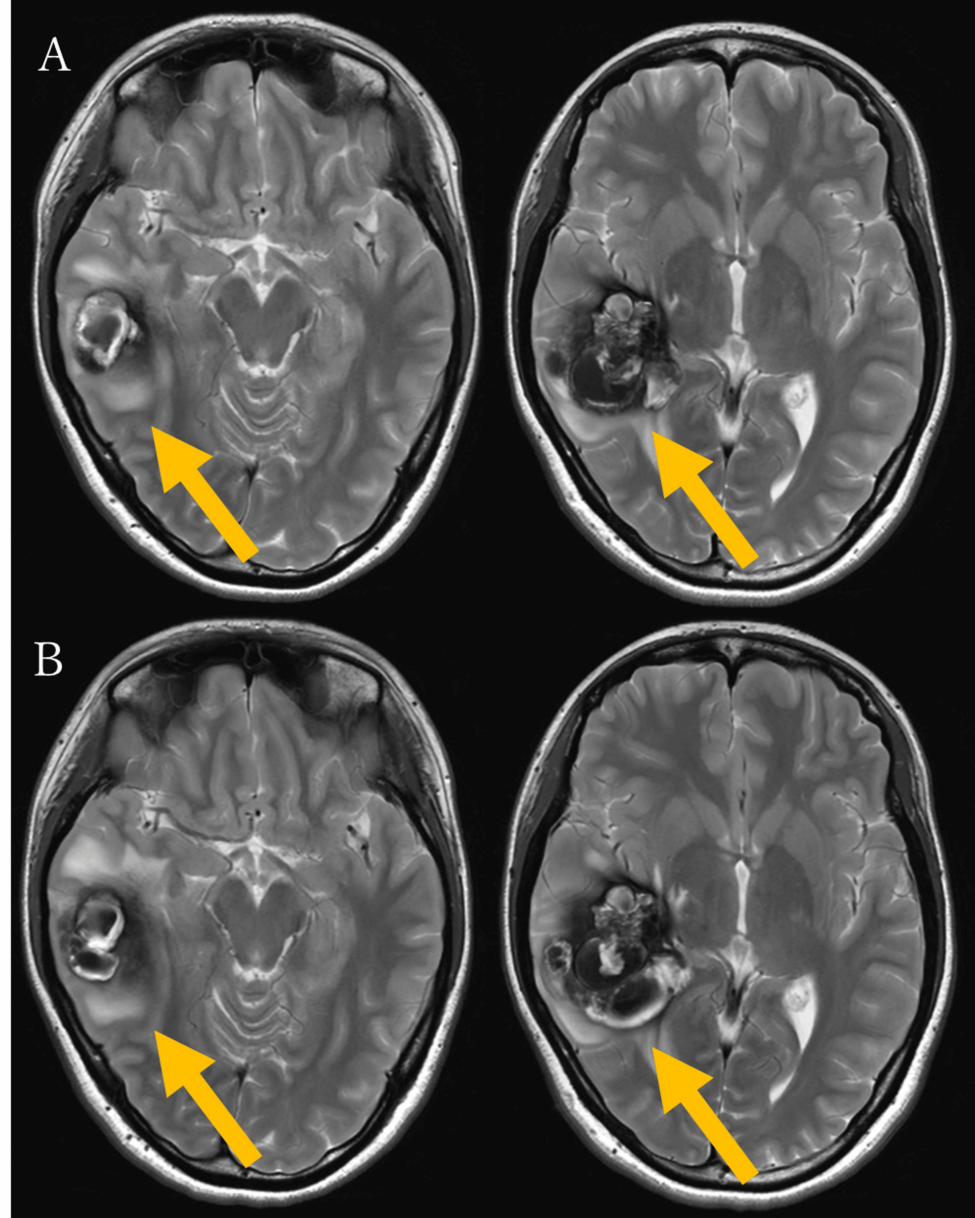
Follow-up brain MRI for the lesion (A) Brain MRI on hospital day 18 (17 days after initial presentation) shows rebleeding and lesion enlargement with a maximum diameter of 37.5 mm on T2-weighted imaging. (B) Brain MRI on day 32 from initial presentation reveals a second rebleeding event and further enlargement of the lesion to 46.8 mm on T2-weighted imaging.

**Figure 4:**
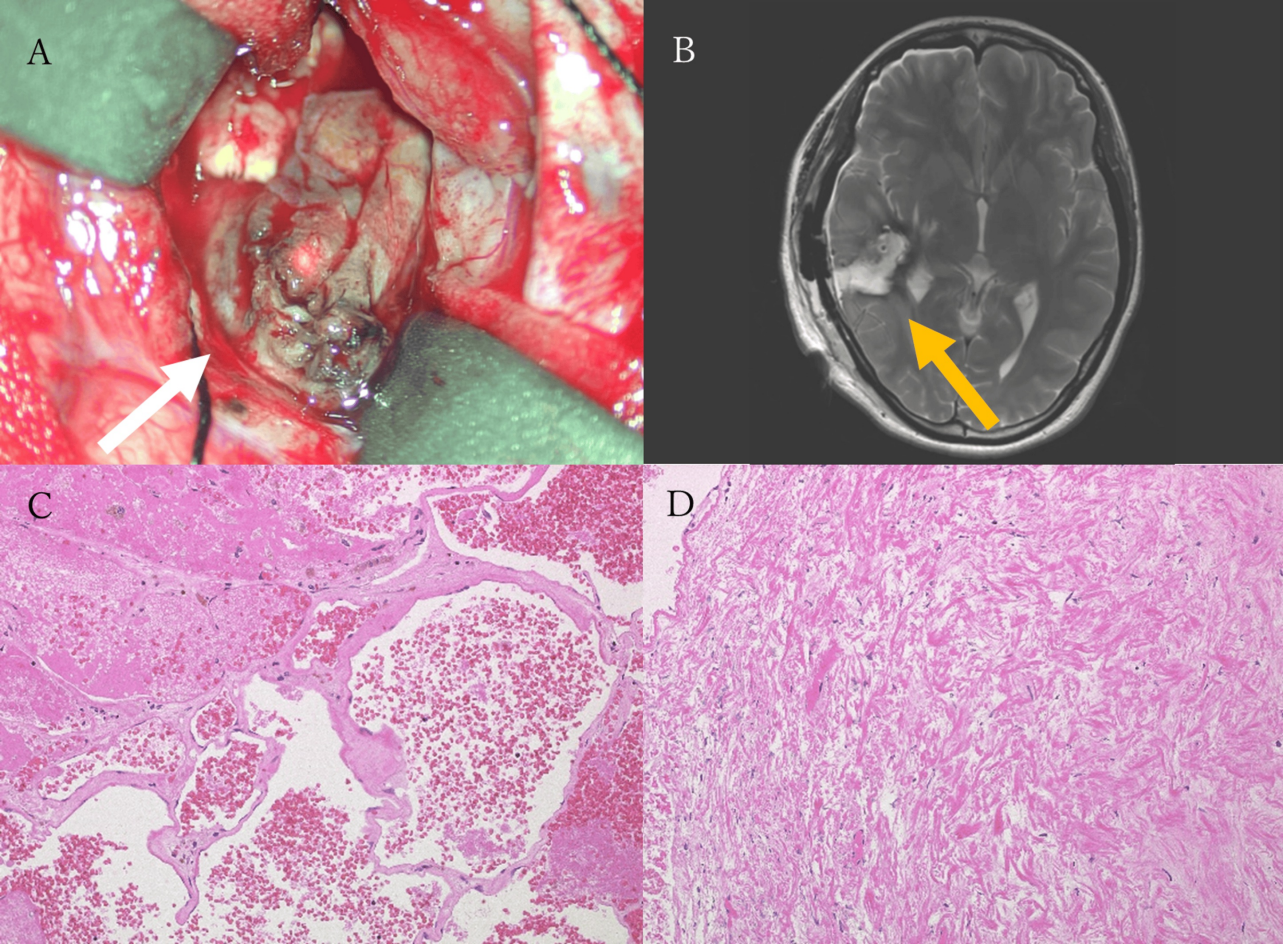
Intraoperative and histopathological findings (A) Intraoperative view shows the cerebral cavernous malformation (CCM); (B) Postoperative axial T2-weighted MRI demonstrates gross total resection of the CCM; (C, D) Hematoxylin and eosin (H&E) staining shows clusters of dilated, thin-walled hyalinized vessels in close apposition, with minimal intervening brain parenchyma. Hemosiderin deposition and surrounding fibrosis are also observed.

Based on these findings, a diagnosis of CCM of the right temporal lobe with associated hemorrhage was made. As this was considered an initial hemorrhage, conservative management was chosen after thorough discussion with the patient. Her symptoms gradually improved. However, a follow-up MRI performed on hospital day 18 revealed rebleeding, with an increase in the lesion’s size to 37.5 mm in maximum diameter (Figure 3A). Despite the rebleeding, the patient remained neurologically intact. Given the absence of neurological deficits and in accordance with the patient’s preference, conservative management was continued. The patient was discharged on hospital day 20 and followed up in the outpatient clinic. On day 32 from initial presentation (13 days post discharge), a follow-up MRI demonstrated a third hemorrhage, with further enlargement of the cavernous malformation to 46.8 mm (Figure 3B). Although the patient remained neurologically intact, likely due to the lesion's location, the lesion exhibited highly aggressive behavior. After a detailed discussion, surgical resection was performed on day 38 from the initial presentation. A craniotomy was performed via the temporal bone, and under navigation guidance, a cortical incision was made directly over the lesion. Intraoperatively, the malformation was found to contain both recent and old hematomas, and it exhibited an elastic consistency. Although caution is generally required to avoid injury to associated DVAs during resection, no such anomaly was observed during surgery, consistent with the findings of preoperative imaging. The malformation was then carefully dissected from the surrounding gliotic tissue and completely resected (Figure 4A). The patient awoke from anesthesia without any new neurological deficits. Postoperative MRI confirmed gross total resection of the lesion (Figure 4B). Histopathological examination confirmed the diagnosis of CCM (Figures 4C, 4D). Her postoperative course was uneventful, and she was discharged on postoperative day 12. Two weeks after discharge, the patient underwent follow-up MRI and clinical evaluation. During this period, no symptoms were reported, and an MRI revealed no evidence of recurrent hemorrhage. The patient remained clinically stable without any complications. As this is a recently treated case, the follow-up period is currently limited. Long-term monitoring is ongoing to assess for potential recurrence or delayed complications.

## Discussion

This report describes the case of a young woman who experienced multiple episodes of intracerebral hemorrhage within a short period and was ultimately diagnosed with a CCM located in the temporal lobe. CCMs are vascular anomalies that are not detectable by angiography. Although often detected incidentally on MRI (in 20%-50% of cases), they can also be discovered during evaluations for seizures (50%), intracerebral hemorrhage (25%), or focal neurological deficits (FND) without overt hemorrhage (25%) [1].

The estimated annual hemorrhage risk in patients without prior bleeding is approximately 0.7%-1.1% per lesion. In contrast, this risk increases significantly to around 4.5% in patients with a history of hemorrhage [2]. The risk of rebleeding is highest during the first two years following the initial hemorrhagic event [2,3]. Additional risk factors for rebleeding include brainstem localization, younger age, and female sex [2,5]. The median interval between the first and second hemorrhages is reported to be eight to 10 months [3,4]. In our case, however, the patient experienced at least two episodes of asymptomatic rebleeding within approximately one month, along with progressive lesion enlargement. This reflects an unusually aggressive clinical course, deviating from the typical natural history of CCMs.

Although rapid rebleeding is uncommon, similar cases have been reported. For instance, Flemming et al. described a case involving a 27-week pregnant woman with a brainstem CCM who experienced a second hemorrhage just two days after the initial bleed, necessitating surgical resection [6]. While pregnancy has traditionally been considered a risk factor for hemorrhage in CCMs [6,7,8], more recent evidence suggests no significant difference in bleeding risk between pregnant and non-pregnant women [9,10]. In our case, a pregnancy test was negative at the time of admission. In another study, Yan et al. reported a 48-year-old woman with endometrial hyperplasia who experienced rapid rebleeding from a CCM [11]. Some studies have proposed that female hormones may influence the biological behavior of these lesions [7,8,11], suggesting that female sex may itself contribute to a more aggressive disease course. Although hormonal levels were not assessed in the present case, measuring female hormone levels may provide valuable insights into the pathophysiological role of hormones in CCMs. Further investigation is warranted to clarify this potential association.

In patients with a third hemorrhagic episode, factors such as brainstem localization, the presence of DVAs, and familial CCMs are associated with an elevated risk of recurrent bleeding [12]. None of these high-risk factors were present in our patient; nevertheless, she experienced three hemorrhagic events within a short timeframe. The first hemorrhage was symptomatic, presenting with headache, nausea, and vomiting, while the second and third episodes were asymptomatic and detected only through serial MRI. Routine MRI surveillance in asymptomatic CCMs remains controversial [13]. However, in symptomatic patients, especially those with a hemorrhagic onset, frequent and careful MRI monitoring appears warranted. In our case, the lesion's temporal lobe location likely spared the patient from neurological deterioration despite repeated bleeding. Nevertheless, recurrent hemorrhages, particularly a third event, have been significantly associated with functional decline and worsening modified Rankin Scale (mRS) scores [12]. This highlights the importance of vigilant imaging follow-up and timely consideration of surgical intervention in select cases. In cases such as the present one, where recurrent hemorrhages occur within a short period, surgical intervention at the time of rebleeding, even if asymptomatic, may significantly reduce the risk of deterioration in patient outcomes.

## Conclusions

Although asymptomatic CCMs are typically managed conservatively, this case illustrates that some lesions may behave aggressively, with repeated hemorrhages and growth occurring silently over a short period. This underscores the potential shortcomings of infrequent imaging, especially in patients with risk factors such as female sex or prior bleeding. To minimize the risk of cumulative neurological impairment, individualized follow-up and consideration of earlier intervention may be warranted, even in the absence of symptoms.
